# Protein Oligomerization

**DOI:** 10.3390/ijms241310648

**Published:** 2023-06-26

**Authors:** Giovanni Gotte, Marta Menegazzi

**Affiliations:** Biological Chemistry Section, Department of Neuroscience, Biomedicine and Movement Sciences, University of Verona, Strada Le Grazie 8, I-37134 Verona, Italy; marta.menegazzi@univr.it

Protein self-association is a biologically remarkable event that involves and affects the structural and functional properties of proteins. Indeed, some proteins, like hemoglobin, display a constitutive quaternary structure, while others can exist as both monomers or as alternative dimeric or oligomeric forms. Controlled self-association, such as the dimerization or larger oligomerization of a protein, can occur spontaneously, constitutively developing into homo- or hetero-derivatives. Alternatively, it can be induced by external events, like macromolecular crowding, phosphorylation, variations in ionic strength, and pH or temperature changes [1]. All of these environmental changes can be desired or undesired phenomena that, in vivo, imply physio-pathological consequences.

When oligomerization is a controlled phenomenon, typically in physiological contexts, it often provides new functions or enhances pre-existing ones. On the other hand, uncontrolled self-association can follow the initial oligomerization origin and lead to pathologic massive aggregation and amyloidogenic fibrillation [2]. Protein oligomers are difficult to precisely define. In controlled self-association, no more than 10–15 protomers are commonly detectable, while uncontrolled aggregation or fibrillation can result in oligomers made up of even 40–50 subunits [3]. Over the past 20–30 years, techniques like chromatography, electrophoresis, immunoblotting, crystallography, and, more recently, Cryo-EM have facilitated a better understanding of the actual monomeric or oligomeric states and the relative behavior of many proteins [4].

In this complex scenario, the Special Issue entitled “Protein Oligomerization” of the *International Journal of Molecular Sciences* aimed to collect contributions that have reported on and analyzed some of the aforementioned aspects. The Issue includes five original articles and three reviews, for a total of eight contributions, each analyzing different aspects related directly, or indirectly, to protein oligomerization. Different proteins were analyzed, and their structural, functional, and computational features were investigated.

In their article, Rezvani Boroujeni et al. [5] report that the 107–120 peptide of the cell-membrane prion protein PrP^c^ plays a protective role in neuroblastoma SH-SY5Y cells against the oligomers of the amyloid beta Aβ_42_ peptide. These oligomers promote the generation of toxic reactive oxygen species (ROS), as well as the cellular uptake of Ca^2+^, which are both responsible for the onset of Alzheimer’s disease. Indeed, the number of peptides or molecules reported to counteract the massive and harmful aggregation of proteins has increased significantly in the last ten to fifteen years. Likewise, there has been an increase in the occurrence of cases of co-morbidity induced by hetero-aggregates composed of different proteins or characterized by fibril polymorphs [2,4]. Therefore, the beneficial interference of a peptide belonging to an essentially PrP^c^ amyloidogenic protein with fibrillogenic Aβ_42_ shows promise in terms of discovering methods to obstruct or slow down protein-deposition-related neurodegenerative diseases.

In a similar context, the contribution of Palazzi et al. [6] elucidates the factors that enable dopamine metabolites to interfere in α-synuclein (α-syn) aberrant aggregation, which is responsible for Parkinson’s disease. The research group recently found the method by which polyphenolic products counteract the amyloidogenic fate of α-syn [7]. In the present case, they instead report that dopamine oxidative derivatives, above all 3,4-dihydroxyphenylacetic acid (DOPAC), inhibit α-syn fibrillation by stabilizing the monomer and restricting its self-association only to dimeric or trimeric states. This effect is associated with the autoxidation of DOPAC, which limits cytotoxicity and the production of ROS. On the other hand, the formation of ROS is favored by large α-syn aggregates. Hence, these findings may contribute to further understand the potentially beneficial biological effects of catecholamine metabolism in combating neurodegenerative diseases related with protein self-association and deposition.

The review contributed by Chen and Rahman [8] discusses the somehow unexpected behavior of the pathogenic mutants of the polyglutamine tract-binding protein 1 (PQBP-1), which are associated with “Repenning syndrome”, a severe X-linked cognitive disorder. Unlike the intrinsically disordered monomeric wt-PQBP-1, these mutants exist as dimers characterized by large folded domains and high thermal stability. While most known neurodegenerative diseases are driven by the early formation of toxic oligomers [9] leading to an uncontrolled and pathogenic aggregation of the monomer, the PQBP-1 mutants form stable dimers that do not progress into massive self-association. Therefore, their toxicity is not ascribable to undesired fibrillation, but rather to their compromised functions compared to the native monomer. The review also recalls and discusses the features of stable dimers of other intrinsically disordered proteins (IDP), and explores the IDP dimerization events involved in protein evolution.

The other articles in the Special Issue do not directly or entirely address pathogenic events.

The review of Gaber and Pavšič [10] presents strategies for modeling and determining the structure of homo-oligomeric proteins. It highlights how internal symmetry is a common feature characterizing the majority of the homo-oligomeric structures. The review explains how symmetry drives the formation of homo-oligomers, and how this feature can be exploited to determine their structure through different experimental approaches, such as X-ray crystallography, NMR, cryo-EM, or computational modelling. Their interface types, as well as their cyclic, dihedral or cubic symmetries were analyzed in detail. The reported literature offers a comprehensive overview of the approaches used to overcome the structural challenges that can arise from protein oligomers.

The review presented by M. Degano [11] focuses on the structure and activity of N-ribohydrolases (NHs), a family of homo-oligomeric enzymes that catalyze the hydrolysis of the N-glycosidic bond in nucleosides and ribosides. Notably, monomeric NHs also exist, but, since they are enzymatically inactive, they serve different physiological roles. In this context, the structural determinants underlying the nature of homo-oligomeric NHs are crucial for tuning their catalytic activity. The author describes the various NHs present in protozoa, bacteria, and plants. Likewise, the NHs structural groups I, II, III and their tetrameric vs. dimeric nature are finely illustrated, providing insights into their biological properties. Additionally, the review presents drug design approaches targeting specific NHs and explores the potential of anti-protozoan vaccination strategies, as well as gene-directed enzyme-prodrug activation therapies.

The remaining three articles in the special issue focus on some secretory pancreatic-type ribonucleases (RNases) [12] and their oligomerization properties [13]. Two articles specifically examine RNase 5, also known as human angiogenin (h-ANG), while the last article describes a peculiar behavior of the oligomers formed by the super-family proto-type RNase A.

The article contributed by Fagagnini et al. [14] analyses the reduced enzymatic ability of three pathogenic h-ANG variants to cleave tRNAs. This limited activity is believed to promote protein aggregation, leading to amyotrophic lateral sclerosis (ALS) or Parkinson’s disease (PD). The mutants are investigated and compared to the wt through multidimensional NMR, UV-CD and dynamic analyses. In addition, the study explores the possibility of h-ANG variants, under oxidizing stress conditions, that allow h-ANG to evade the cellular RNase inhibitor [15], to induce the production of tiRNA fragments capable of tetramerization, and to form G-quadruplex structures. G-quadruplexes are thought to promote stress-granule formation, inhibiting protein synthesis and undesired aggregation, thereby supporting the survival of motor neurons. Thus, the reduced tRNA degradation by h-ANG mutants should reduce the amount of tRNA and G-quadruplex. However, this work does not provide conclusive evidence of G-quadruplex formation in both wt and h-ANG mutants. Furthermore, the wt and the mutants exhibit similar structures and rigidity, suggesting that new investigations are needed to further clarify the precise role of h-ANG mutations in the mentioned diseases.

The other article focused on h-ANG, by Fasoli et al. [16], reports that the wt and two out of three different pathogenic mutants are capable of dimerization through the mechanism called 3D domain swapping (3D-DS). Notably, this mechanism is common with many proteins directly involved in amyloidogenic deposition diseases [17]. Given the implication of h-ANG in both ALS and PD, the authors aimed to investigate if the mutants and the wt displayed different abilities to self-associate. The results indicate, for the first time, that h-ANG can dimerize through the swapping of its N-termini, but does not undergo massive aggregation, although two h-ANG mutants exhibit a higher extent of dimerization than the wt. However, the inability to overcome dimerization, likely due to the impossibility to additionally swap its C-terminus [16,17], suggests again that h-ANG mutants act in neurodegenerative diseases through defects affecting their functional roles, or interactions with receptors or membranes, rather than through massive auto-aggregation.

Finally, the article of Gotte et al. [18] reports the peculiar behavior displayed by some of the oligomers that RNase A forms through the mentioned 3D-DS mechanism [13]. After storage at 4 °C for about one year, certain oligomers, separated from the others, formed detectable amounts of very large aggregates, referred to as “super-aggregates” (SAs), along with low-soluble derivatives. Notably, only the oligomeric species containing at least one N-swapped subunit can evolve into SAs, while those exclusively formed by C-terminal end swapping, as well as the monomer, do not. A recent study by Noji et al. discovered that the RNase A monomer can generate amyloid derivatives upon the breakdown of the super-saturation barrier [19]. Instead, Gotte et al. found that SAs derive only from RNase A N-swapped oligomers, and investigations of these species with WB, Thioflavin T (ThT) fluorescence and TEM assays do not indicate the presence of fibrillary derivatives, although SAs display partial ThT positivity. Nonetheless, despite behaving as an “autochaperone” blocking extensive aggregation [20], RNase A confirms here to be potentially prone to fibrillation, and the paper proposes a tentative model to describe how SAs may form.

As mentioned at the beginning, it is noteworthy that each one of the eight contributions included in the Special Issue analyzes and highlights different aspects of the “Protein Oligomerization” topic. Two articles directly investigate pathologies related to oligomer-induced amyloidosis [5,6], while three others examine proteins or pathogenic variants involved in neurodegenerative diseases, but not inherently exhibiting amyloidosis [8,14,16]. Additionally, one review provides detailed insights into the structural and functional features of the oligomeric family of N-Ribohydrolases [11], while another review lists and exhaustively describes various approaches and methods useful for modeling and determining the structure of homo-oligomeric proteins in general [10]. Finally, another paper reveals the peculiar long-term evolution behavior of RNase A 3D-DS oligomers [18], highlighting again how protein 3D-DS oligomerization could represent a preliminary, sometimes necessary, step favoring the uncontrolled aggregation of proteins.

Therefore, the editors greatly appreciate all the efforts of every author involved, as their valuable contributions have enriched this Special Issue. Although the presented contributions analyzed different proteins undergoing various oligomerization events, and mechanisms, described different experimental approaches, and have certainly expanded the readers’ knowledge, the topic of protein oligomerization has the potential to gather a large number of articles and reviews. This possibility can serve as motivation to perform further investigations.
